# Qingre–Huatan–Liqi formula attenuates FPM-induced lung injury via modulation of MAPK signaling and NETs formation

**DOI:** 10.3389/fphar.2026.1778590

**Published:** 2026-06-24

**Authors:** Jie Yang, Yaqian Liu, Yufeng Meng, Hongtao Wang, Xianqiang Zhou, Xuyi Zhao, Cuiling Feng

**Affiliations:** 1 Department of Traditional Chinese Medicine, Peking University People’s Hospital, Beijing, China; 2 Department of Integration of Chinese and Western Medicine, School of Basic Medical Sciences, Peking University, Beijing, China; 3 Fuzhou University Affiliated Provincial Hospital, Fuzhou, China

**Keywords:** FPM, lung injury, MAPK14/p38 MAPK, NETs, oxidative stress

## Abstract

**Background:**

Fine particulate matter (PM2.5, FPM) can induce and exacerbate chronic obstructive pulmonary disease (COPD) by triggering oxidative stress and multiple other pathogenic pathways. Our previous studies have demonstrated that Qingre–Huatan–Liqi formula (QRHTLQ) can improve the symptoms of patients with acute exacerbation of COPD (AECOPD), and animal experiments have suggested that QRHTLQ may prevent AECOPD via the EGFR–PI3K–AKT pathway. However, the protective effects and precise mechanisms by which QRHTLQ attenuates FPM-induced lung injury remain unclear.

**Methods:**

FPM-induced lung injury models were established by repeated intratracheal instillations of FPM every 3 days. Thirty rats were randomly divided into control, model, low-dose QRHTLQ, medium-dose QRHTLQ, high-dose QRHTLQ, and erythromycin groups. The therapeutic effects of QRHTLQ were evaluated by H&E staining and qPCR. LC–MS was used to identify blood-absorbed active constituents of QRHTLQ. Proteomics combined with network pharmacology was applied to identify key targets. The effects of QRHTLQ on these targets and related processes were validated by immunofluorescence, ELISA, and Western blot. Molecular docking and molecular dynamics simulations were performed to evaluate constituent–target binding and complex stability. *In vitro*, dHL-60 cells were stimulated with FPM to explore the intervention effects of QRHTLQ-containing serum and baicalin.

**Results:**

QRHTLQ alleviated FPM-triggered airway inflammation, downregulated pulmonary IL-6, IL-1β, TNF-α mRNA as well as ROS levels in bronchoalveolar lavage fluid (BALF). Six major blood-absorbed active constituents were identified using LC–MS. Integrated analyses suggested that QRHTLQ interferes with neutrophil extracellular traps (NETs) formation, with MAPK14 (P38α, the major isoform of P38 MAPK) identified as a core target. QRHTLQ decreased PAD4 in BALF and lung tissue, reduced NETs levels in lung tissue, and decreased the p-P38/P38 ratio. Baicalin exhibited the most favorable binding energy with MAPK14, and molecular dynamics simulations demonstrated that the baicalin–P38 MAPK14 complex was structurally stable. *In vitro* experiments confirmed that baicalin significantly reduced NETs release in dHL-60 cells and downregulated the p-P38/P38 ratio, thereby inhibiting MAPK14 activation.

**Conclusion:**

QRHTLQ alleviates FPM-induced lung injury by modulating MAPK signaling and inhibiting NETs formation. Baicalin is likely a key blood-absorbed active component through which QRHTLQ exerts these protective effects.

## 1 Introduction

The continuously increasing concentration of fine particulate matter (FPM) in the environment has become one of the major causes of air pollution, and the respiratory system is the primary target system affected by FPM (Chen and Hoek, 2020; Park et al., 2021). Long-term inhalation exposure to FPM not only leads to persistent airway inflammation and structural damage to lung tissue but also promotes the onset and progression of chronic respiratory diseases such as COPD, increases the risk of acute exacerbations, and seriously impairs respiratory health and quality of life (Bo et al., 2021; Li et al., 2022; Delavar et al., 2023).

Neutrophilic inflammation plays an important role in FPM-induced pulmonary inflammation. On the one hand, upon invading the lungs, FPM and its carried toxic components rapidly recruit neutrophils to migrate toward the injured sites, which then exert an initial defensive effect by phagocytosing particulate matter and releasing antimicrobial substances. On the other hand, sustained FPM exposure can over-activate neutrophils, triggering them to initiate the extracellular trap formation program. Excessive NETs not only directly damage pulmonary epithelial and vascular endothelial cells, exacerbate local inflammatory responses (Keir and Chalmers, 2022) and tissue fibrosis (Suzuki et al., 2020), but also disrupt the body’s immune homeostasis, thereby further amplifying the persistent lung injury caused by FPM (Grabcanovic-Musija et al., 2015). The current clinical management of FPM-induced lung injury focuses primarily on treating its complications, such as emphysema and COPD, with bronchodilators, expectorants and inhaled glucocorticoids (Singh et al., 2019). At that stage, lung tissue damage is often irreversible, and little attention is given to early interventions targeting oxidative stress and dysregulated inflammatory networks during the initial phase of FPM-induced lung injury. Therefore, targeted intervention of neutrophilic inflammation and NETs release at an early stage, before disease complications develop, may play a crucial role in alleviating FPM-induced lung injury.

Traditional Chinese medicine (TCM) adheres to the principle of “treating disease before its onset”, emphasizing preventive interventions prior to disease development to halt its occurrence. Furthermore, a growing number of TCM formulas have demonstrated tremendous potential in respiratory diseases (Zhao et al., 2025; Chen et al., 2026). QRHTLQ, also known as “Louqin Zhisou”, originates from the prescription “Qingjin Huatan decoction” recorded in the Ming dynasty classic *Yixue Tongzhi*. It is composed of *Trichosanthes kirilowii* Maxim., *Scutellaria baicalensis* Georgi, *Rhaponticum uniflorum* (L.) DC., *Forsythia suspensa* (Thunb.) Vahl, *Fritillaria thunbergii* Miq., *Pinellia ternata* (Thunb.) Makino, *Peucedanum praeruptorum* Dunn and *Platycodon grandiflorus* (Jacq.) A. DC. As an empirical prescription in TCM practice, QRHTLQ has been widely used clinically for the treatment of acute exacerbation of COPD (AECOPD). Our previous clinical findings showed that QRHTLQ can relieve symptoms in AECOPD patients and reduce the frequency of cough, sputum production and wheezing (Feng and Yu, 2011). In a rat model established by the intratracheal instillation of LPS combined with chronic cigarette smoke exposure, we further demonstrated that QRHTLQ ameliorates AECOPD via the EGFR–PI3K–AKT signaling pathway (Feng et al., 2019). However, the composition of ambient fine FPM is substantially more complex than that of cigarette smoke. Upon invading the lungs, FPM is more prone to triggering intense, acute neutrophilic inflammation and inducing the excessive release of NETs. This distinct pathological mechanism differs significantly from the bacterial stress induced solely by LPS or the epithelial injury caused by smoke. Previous studies on QRHTLQ were confined to its protective effects on epithelial pathways, leaving its potential to regulate neutrophil homeostasis unclarified. Furthermore, as a complex TCM formula, its *in vivo* blood-absorbed active constituents and precise molecular targets against FPM-induced lung injury remain completely unknown.

In this study, we established a FPM-induced lung injury model via periodic intratracheal instillation of FPM. Based on the biological process of NETs and the key target of MAPK14 (P38α, the major isoform of P38 MAPK), which were identified by proteomics and network pharmacology, we systematically investigated the potential mechanisms and bioactive components underlying the intervention effect of QRHTLQ on FPM-induced lung injury.

## 2 Materials and methods

### 2.1 Preparation of QRHTLQ

QRHTLQ consists of eight medicinal materials: Trichosanthis Pericarpium (Gualoupi), Scutellariae Radix (Huangqin), Rhapontici Radix (Loulu), Forsythiae Fructus (Lianqiao), Fritillariae Thunbergii Bulbus (Zhebeimu), Pinelliae Rhizoma (Banxia), Peucedani Radix (Qianhu), and Platycodonis Radix (Jiegeng). The formula was uniformly manufactured by Beijing Kangrentang Pharmaceutical Co., Ltd. Based on equivalent clinical dosages for adults, the herbs were processed into granules at a fixed crude-herb ratio of 15:10:10:12:10:9:10:10. The granules were subsequently prepared for gavage according to the body weights of the rats. The specific composition and dosage of each component are presented in Table 1.

**TABLE 1:** Composition and drug dosage of the QRHTLQ prescription.

Formula	Herbs	Adult herbal dosage (g/d)	Herbal medicine: Granules	Dosage of granules for rats (g/kg/d*bw)
QRHTLQ	Trichosanthis pericarpium	15	5:1	0.315
	Scutellariae radix	10	10:1	0.105
	Rhapontici radix	10	20:1	0.0525
	Forsythiae fructus	12	10:1	0.126
	Fritillariae thunbergii bulbus	10	10:1	0.105
	Pinelliae rhizoma	9	10:1	0.0945
	Peucedani radix	10	10:1	0.105
	Platycodonis radix	10	5:1	0.21

### 2.2 Establishment of FPM-induced pulmonary injury model in rats and drug administration

Specific pathogen-free (SPF) Wistar rats, aged 6–8 weeks and weighing 180–220 g, were purchased from Beijing Vital River Laboratory Animal Technology Co., Ltd. FPM was purchased from the National Institute of Standards and Technology (SRM 1649b) and was used as a standard reference particulate matter to establish a PM2.5-like lung injury model. The animals were housed under controlled conditions (temperature 23 °C–26 °C, relative humidity 40%–65%, 12 h light/dark cycle) with *ad libitum* access to food and water. Erythromycin was selected as a positive control due to its established anti-inflammatory and neutrophil-suppressing effects. After 1 week of acclimatization, the rats were randomly assigned to one of six groups (n = 5 rats per group) using a random number table: the control group, the FPM model group, the FPM + QRHTLQ low-dose (QRHTLQ-L), QRHTLQ medium-dose group (QRHTLQ-M), QRHTLQ high-dose group (QRHTLQ-H), and the FPM + erythromycin positive control group.

The FPM dose was calculated based on the heavy air pollution threshold (150 μg/m^3^) and the daily respiratory volume of rats (0.144 m^3^), yielding a daily inhalation dose of 21.6 μg. To match the administration frequency of once every 3 days, the single intratracheal instillation dose was determined to be a 3-day cumulative amount of 65 μg. All groups except for the control group received intratracheal instillations of FPM at a dosage of 65 μg per administration (prepared as a 325 μg/mL suspension) after anesthesia with isoflurane (3%–5%, inhaled) once every 3 days for a total of 10 instillations. Following the instillation, the rats were secured on a rat board and gently rotated for 1 min to facilitate the uniform distribution of particles into the distal airways. The control group received an equal volume of PBS at the same frequency as a sham procedure.

From the first day of modeling, the rats in the treatment groups were intragastrically administered QRHTLQ or erythromycin once daily (vehicle: PBS; volume: 10 mL/kg). Based on the human-equivalent clinical dose, the medium dose of QRHTLQ was 1113 mg/kg/day, the high dose was twice that amount, and the low dose was half that dose. The erythromycin group received 100 mg/kg/day. The rats in the control and model groups received an equivalent volume of PBS. On the day following the final FPM instillation, the rats were euthanized via intraperitoneal injection of sodium pentobarbital (100 mg/kg, i. p.), and samples were subsequently collected. All experimental procedures were approved by the Experimental Animal Ethics Committee of Peking University People’s Hospital (No. 2020PHE092) and were performed in strict accordance with the ARRIVE guidelines.

### 2.3 Histopathology and immunofluorescence staining

Lung tissues were fixed with 4% paraformaldehyde for 48 h, dehydrated, and embedded in paraffin. H&E staining was performed to evaluate histopathological changes (Henderson et al., 1996). For immunofluorescence (IF), lung sections underwent deparaffinization and heat-induced antigen retrieval in citrate buffer. For *in vitro* IF, dHL-60 cells were fixed with 4% paraformaldehyde and permeabilized with 0.2% Triton X-100. Both tissue sections and cells were blocked for 1 h and incubated overnight at 4 °C with primary antibodies: CitH3 (1:200), MPO (1:200) and DAPI for tissue; NE (1:200), CitH3 (1:200) and SYTOX Green for cells (Brinkmann et al., 2016).

### 2.4 ELISA and qPCR

Bronchoalveolar lavage fluid (BALF) was centrifuged at 3000 rpm for 15 min, after which the supernatant was collected. The levels of PAD4 (Wuhan Saipei, SP13578), CitH3 (CIOBO BIO, CB12261-Ra), and ROS (CIOBO BIO, CB10450-Ra) in the BALF were measured using ELISA kits. A total of 30 mg of lung tissue was processed with TRIzol reagent to extract RNA, which was reverse transcribed into cDNA for the quantitative PCR analysis of IL-1β, IL-6, and TNF-α mRNA expression. The sequences of the primers used in this study are listed in Table 2. The qPCR amplification was performed under the following conditions: an initial denaturation at 94 °C for 5 min, followed by 40 cycles of 94 °C for 30 s, 58 °C for 30 s, and 72 °C for 30 s. Relative mRNA expression levels were normalized to GAPDH and calculated using the 2^-ΔΔCt^ method.

**TABLE 2:** qPCR primers.

Gene	Forward primer	Reverse primer
IL-6	TCC​TAC​CCC​AAC​TTC​CAA​TGC​TC	TTG​GAT​GGT​CTT​GGT​CCT​TAG​CC
TNF-α	AAA​TGG​GCT​CCC​TCT​CAT​CAG​TTC	TCT​GCT​TGG​TGG​TTT​GCT​ACG​AC
IL-1β	CAC​CTC​TCA​AGC​AGA​GCA​CAG	GGG​TTC​CAT​GGT​GAA​GTC​AAC
GAPDH	GGC​ACA​GTC​AAG​GCT​GAG​AAT​G	ATG​GTG​GTG​AAG​ACG​CCA​GTA

### 2.5 Identification of blood-absorbed components of QRHTLQ by LC–MS

QRHTLQ-containing serum was centrifuged at 14,000 rpm for 5 min and subsequently filtered through a 0.22 μm membrane. The mass spectrometry analysis was performed using a Thermo Fisher Q Exactive Plus high-resolution LC–MS system operated in both positive and negative ion modes.

### 2.6 Network pharmacology and bioinformatics analyses

The blood-absorbed components of QRHTLQ identified by LC–MS were screened in the TCMSP database with the criteria of oral bioavailability (OB) ≥ 30% and drug likeness (DL) ≥ 0.18. The major active compounds that met these criteria were subjected to target prediction and network pharmacology analyses. Cytoscape 3.10 was used for the weighted visualization of the compound–target interactions. The keyword “PM2.5 lung damage” was used to retrieve genes associated with FPM-induced pulmonary injury from the GeneCards database. A functional enrichment analysis of the related proteins was conducted using the DAVID database, with *p* < 0.05 considered to indicate statistical significance.

### 2.7 Proteomics

Lung tissues were lysed and ultrasonically disrupted to extract total proteins, which were quantified using the bicinchoninic acid (BCA) assay. Proteins were reduced, alkylated, and enzymatically digested with trypsin into peptides, followed by desalting and purification on a C18 column and subsequent LC–MS analysis. Raw data were processed using Proteome Discoverer 2.2 and Skyline for protein identification and label-free proteomic quantification. Differentially expressed proteins were screened based on |log_2_FC| ≥ 0.58 and a *p* < 0.05. The enrichment analysis of differentially expressed proteins between groups was performed using the DAVID database, and the proteomic results were visualized using Python.

### 2.8 Western blot

Lung tissues were homogenized in RIPA lysis buffer, followed by centrifugation at 13,000 rpm for 15 min. The protein concentration in the supernatant was determined using a BCA assay. Equal amounts of protein were separated by SDS–PAGE and transferred onto PVDF membranes, which were blocked with 5% skim milk for 1 h. The membranes were incubated overnight at 4 °C with primary antibodies against PAD4 (abclonal A16188, 1:1000), P38 MAPK (CST 9212T, 1:1000), phosphorylated P38 (p-P38, CST 4511T, 1:1000), MPO (Proteintech 66177-1-Ig, 1:1000), and GAPDH (Proteintech 10494-1-AP, 1:3000), followed by an incubation with the appropriate secondary antibodies the following day. A semiquantitative analysis of band intensities was performed using ImageJ.

### 2.9 Molecular docking and molecular dynamics simulation

Key blood-absorbed compounds of QRHTLQ identified through LC–MS combined with network pharmacology, along with their core protein targets determined by proteomic and bioinformatic analyses, were subjected to molecular docking and molecular dynamics simulation. Molecular docking was performed using AutoDock Vina, and the resulting protein–ligand complexes were visualized using PyMOL (Trott and Olson, 2010).

Molecular dynamics simulations were performed using GROMACS 2022 for a total duration of 100 ns. The CHARMM36 force field was applied for protein parametrization, while the ligand topologies were generated using the CGenFF force field. The protein–ligand complexes were placed in a cubic simulation box under periodic boundary conditions. The box was solvated with water molecules using the TIP3P water model, with a minimum distance of 1.2 nm between the solute and the boundary. Long-range electrostatic interactions were treated using the particle mesh Ewald (PME) method, and the Verlet cutoff scheme was applied. The system underwent equilibration under the NVT ensemble followed by the NPT ensemble for 100,000 steps each (100 ps) using a coupling constant of 0.1 ps. Both van der Waals and Coulomb interactions were calculated with a cutoff distance of 1.0 nm. Finally, production MD simulations were conducted at a constant temperature of 310 K and pressure of 1 bar for 100 ns using GROMACS 2022 (Pronk et al., 2013).

### 2.10 Preparation of QRHTLQ-containing serum

Healthy SPF Wistar rats were randomly divided into a blank control serum group and a QRHTLQ serum group. Rats in the QRHTLQ serum group were continuously administered QRHTLQ via intragastric gavage at a high dose (2226 mg/kg/day) for 1 week. The blank control serum group received an equal volume of PBS. Two hours after the final administration, the rats were deeply anesthetized with inhaled isoflurane, and whole blood was collected via the abdominal aorta. The collected blood was allowed to clot at room temperature for 1–2 h and subsequently centrifuged at 3000 rpm for 15 min at 4 °C to isolate the serum. The obtained supernatant was heat-inactivated in a 56 °C water bath for 30 min to eliminate complement interference, followed by sterilization through a 0.22 μm membrane filter. The prepared serum was aliquoted and stored at −80 °C for subsequent *in vitro* cell experiments.

### 2.11 Induction and subculture of dHL-60 cells

HL-60 cells were obtained from Boster Biological Technology Co., Ltd. After thawing, cells were seeded uniformly into 10 cm culture dishes containing RPMI 1640 complete medium and subcultured at a ratio of 1:2 or 1:3 every 2 days based on cell density. For differentiation, HL-60 cells were treated with 1.25% DMSO for 5 days to generate differentiated HL-60 (dHL-60) cells.

### 2.12 Cell viability assay

dHL-60 cells were treated with 10% QRHTLQ-containing serum. After incubation at 37 °C for 4 h, the CCK-8 assay kit was used to detect the effects of 10% QRHTLQ-containing serum on the viability of dHL-60 cells.

In addition, dHL-60 cells were treated with gradient concentrations of baicalin at final concentrations of 0, 20, 50, 100 and 200 μM. The blank control group was supplemented with an equal volume of PBS. Following incubation at 37 °C for 2 h, the CCK-8 assay kit was applied to evaluate the effect of baicalin on dHL-60 cell viability.

### 2.13 Screening of FPM intervention dose and stimulation protocol for dHL-60 cells

dHL-60 cells were treated with FPM at concentrations of 25, 50, 100 and 200 μg/mL respectively and incubated for 4 h. The supernatant was collected to detect the extracellular dsDNA level, so as to explore the optimal concentration of FPM for inducing NETs production in dHL-60 cells.

dHL-60 cells were divided into three groups: control group, model group and QRHTLQ-containing serum group. The control group was cultured normally for 4 h. The model group was stimulated with the optimal concentration of FPM for 4 h. The medium of the drug-containing serum group was supplemented with QRHTLQ-containing serum, while the control and model groups were supplemented with blank rat control serum. Both the model group and QRHTLQ-containing serum group were stimulated with the optimal concentration of FPM for 4 h.

Furthermore, cells were divided into another three groups: control group, model group and baicalin group. Cells in the model group and baicalin group were stimulated with the optimal concentration of FPM for 4 h, among which the baicalin group was pretreated with 100 μM baicalin for 2 h.

After the above cell stimulation, the supernatant and cell pellet of each group were collected separately for subsequent related detection.

### 2.14 Analysis

All data were analyzed using GraphPad Prism 9.0 and are presented as mean ± SD. Comparisons among multiple groups were performed using ordinary one-way ANOVA followed by Dunnett’s multiple-comparison test. A *p* value <0.05 was considered statistically significant.

## 3 Results

### 3.1 QRHTLQ alleviates FPM-induced airway injury

**FIGURE 1:**
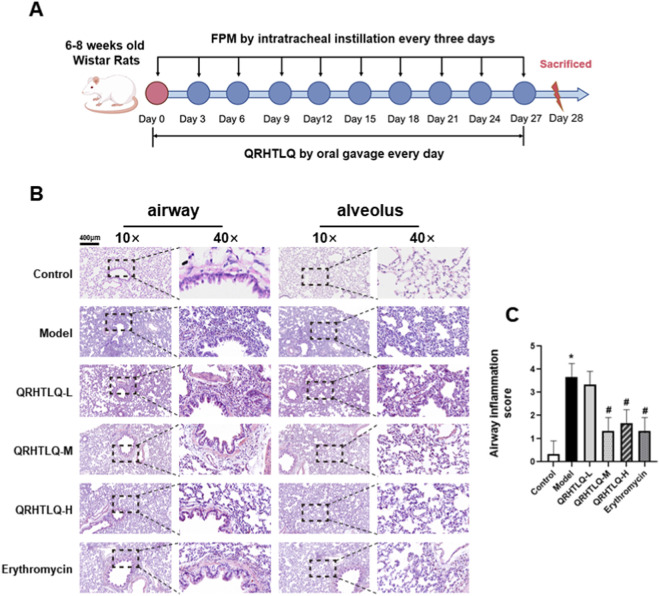
QRHTLQ attenuates FPM-induced airway injury. **(A)** Animal model of lung injury induced by FPM. Starting from the first day of the experiment, the treatment groups were administered the corresponding doses of QRHTLQ, erythromycin, or PBS by oral gavage. Animals in the model group and the drug administration groups received intratracheal instillation of 65 μg FPM once every 3 days. **(B)** Representative images of H&E-stained lung sections showing the protective effects of QRHTLQ against FPM-induced pathological changes. **(C)** Airway inflammation scores (n = 3). #*p* < 0.05 vs. Model; *p < 0.05 vs. Control.

To investigate the intervention effects of QRHTLQ on FPM-induced lung injury, we performed H&E staining on lung tissue sections from each group. H&E staining revealed that the intratracheal instillation of FPM induced marked pathological alterations in rat lung tissue, characterized by extensive inflammatory cell infiltration around the airways, thickening of the alveolar septa, and alveolar fusion. QRHTLQ treatment markedly attenuated these pathological changes and significantly reduced airway inflammation scores (Figures 1B,C, p < 0.05), suggesting that QRHTLQ exerts a certain protective effect against FPM-induced lung injury.

### 3.2 QRHTLQ attenuates FPM-induced increases in BALF ROS levels and pulmonary inflammatory cytokine mRNA expression

**FIGURE 2:**
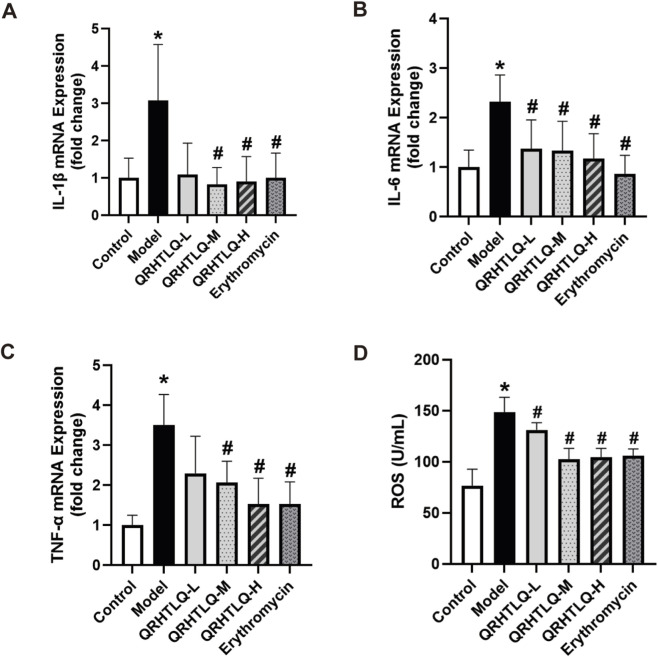
QRHTLQ can reduce the pulmonary expression of inflammatory factors and oxidative stress caused by FPM. **(A–C)** Expression levels of the IL-1β, IL-6, and TNF-α mRNAs in lung tissue, as determined by qPCR (n = 5). **(D)** The ROS content was detected using ELISA (n = 5). #*p* < 0.05 vs. Model; *p < 0.05 vs. Control.

To further evaluate the anti-inflammatory and anti-oxidative properties of QRHTLQ, we quantified the expression of pro-inflammatory cytokines (IL-1β, TNF-α, and IL-6) in lung tissues via qPCR, and ROS in BALF was determined by ELISA to assess the oxidative stress response. Quantitative PCR analysis demonstrated that FPM exposure significantly upregulated the mRNA expression levels of IL-1β, TNF-α, and IL-6 in lung tissue, whereas QRHTLQ treatment reduced their expression in a dose-dependent manner (Figures 2A–C). The positive control, erythromycin, also suppressed the expression of these proinflammatory cytokines. These findings indicate that QRHTLQ effectively alleviates FPM-induced pulmonary inflammation. Consistent with this observation, the ELISA results showed that the intratracheal instillation of FPM markedly increased ROS levels in the BALF, suggesting that oxidative stress was induced in the lung tissue. Compared with the model group, the groups treated with both QRHTLQ and erythromycin presented significantly lower ROS levels (*p* < 0.05) (Figure 2D). These results suggest that QRHTLQ can counteract FPM-induced pulmonary inflammation and alleviate oxidative stress.

### 3.3 Six key blood-absorbed components of QRHTLQ were identified using LC‒MS

**FIGURE 3:**
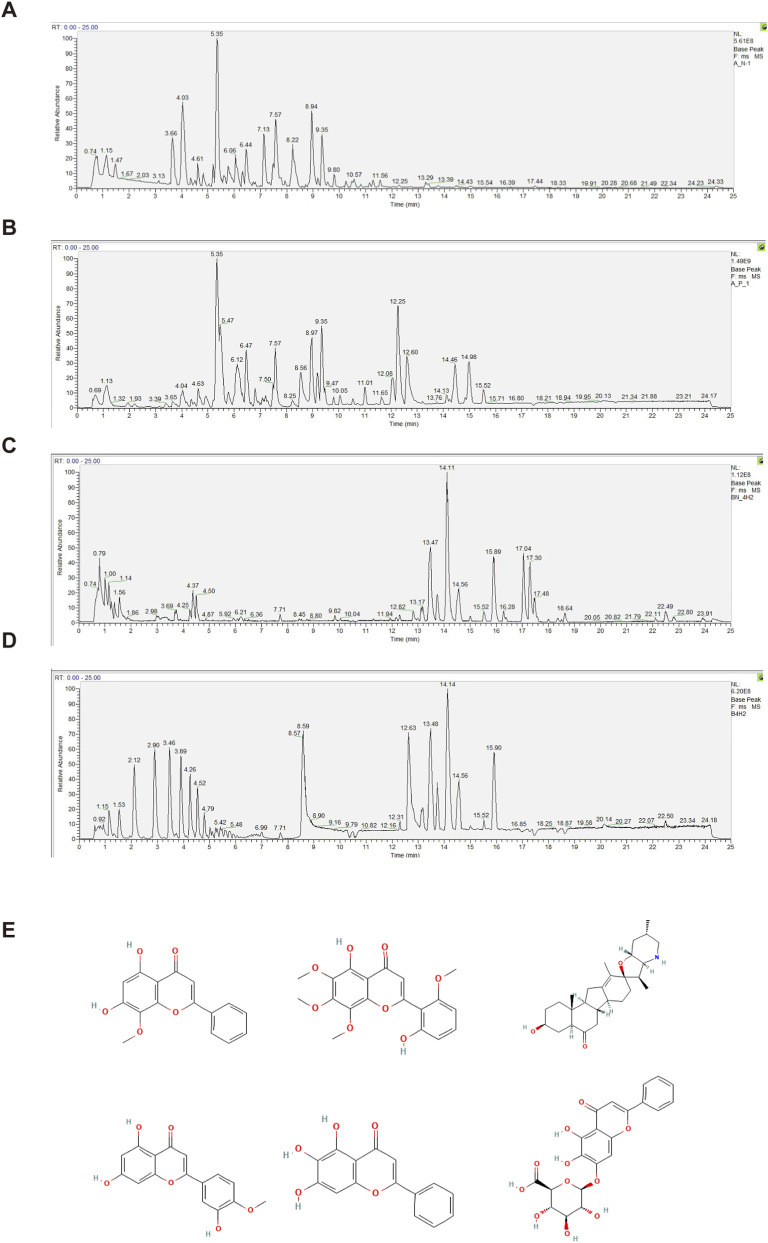
LC–MS characterization of QRHTLQ and identification of blood-absorbed components. **(A,B)** Representative base-peak chromatograms of QRHTLQ recorded using LC–MS in positive-ion **(A)** and negative-ion **(B)** modes. **(C,D)** Base-peak chromatograms of drug-containing rat serum collected after the oral administration of QRHTLQ detected in positive-ion **(C)** and negative-ion **(D)** modes. **(E)** Chemical structures of representative blood-absorbed components identified by LC–MS.

To identify the specific potential substances through which QRHTLQ intervenes in FPM-induced lung injury, we analyzed QRHTLQ and its blood-absorbed components using LC-MS. Based on the retention time, accurate mass measurements, and MS/MS fragmentation patterns, a total of 54 major components were identified in the QRHTLQ preparation (Table 3; Figures 3A,B), and 31 blood-absorbed components were identified in QRHTLQ-containing serum (Table 4; Figures 3C,D). Further screening of the blood-absorbed components using OB and DL parameters from the TCMSP database resulted in the selection of six major active blood-absorbed compounds, which were subsequently used for the network pharmacology analysis (Figure 3E).

**TABLE 3:** Major chemical constituents of QRHTLQ.

Metabolite name	Formula	Ion	Adduct type
Vanillic acid	C_8_H_8_O_4_	N	[M-H]^-^
Ursolic acid	C_30_H_48_O_3_	N/P	[M-H]^-^/[m+H-H2O]^+^
Succinic acid	C_4_H_6_O_4_	N	[M-H]^-^
Skullcapflavone II	C_19_H_18_O_8_	N/P	[M-H]^-^/[m+h]^+^
Secoisolariciresinol	C_20_H_26_O_6_	N	[M-H]^-^
Scopoletin	C_10_H_8_O_4_	N/P	[M-H]^-^/[m+h]^+^
Resveratrol	C_14_H_12_O_3_	N	[M-H]^-^
Quercetin	C_15_H_10_O_7_	N	[M-H]^-^
Pinoresinol	C_20_H_26_O_6_	N	[M-H]^-^
Phillyrin	C_27_H_34_O_11_	N	[M + FA-H]^-^
Phenylalanine	C_9_H_11_NO_2_	N/P	[M-H]^-^/[m+h]^+^
Palmitic acid	C_16_H_32_O_2_	N	[M-H]^-^
Oroxindin	C_22_H_20_O_11_	N	[M-H]^-^
Oleic acid	C_18_H_34_O_2_	N	[M-H]^-^
Forsythoside B	C_34_H_44_O_19_	N	[M-H]^-^
Ferulic acid	C_10_H_10_O_4_	N	[M-H]^-^
Chrysin	C_15_H_10_O_4_	N	[M-H]^-^
Catechol	C_6_H_6_O_2_	N	[M-H]^-^
Baicalin	C_21_H_18_O_11_	N	[M-H]^-^
Baicalein	C_15_H_10_O_5_	N	[M-H]^-^
Asiatic acid	C_30_H_48_O_5_	N	[M-H]^-^
Adenine	C_5_H_5_N_5_	N/P	[M-H]^-^/[m+h]^+^
Umbelliferone	C_9_H_6_O_3_	N/P	[M-H]^-^, [m+h]^+^
Sucrose	C_12_H_22_O_11_	N	[M-H]^-^
Homogentisic acid	C_8_H_8_O_4_	N	[M-H]^-^
Hirsutrin	C_21_H_20_O_12_	N	[M-H]^-^
Caffeic acid	C_9_H_8_O_4_	N/P	[M-H]^-^, [m+h]^+^
Wogonin	C_16_H_12_O_5_	P	[M + H]^+^
Trigonelline	C_7_H_7_NO_2_	P	[M + H]^+^
Thymol	C_10_H_14_O	P	[M + H]^+^
Sinapyl alcohol	C_11_H_14_O_4_	P	[M + Na]^+^
Salidroside	C_14_H_20_O_7_	P	[M + NH_4_]^+^
Psoralen	C_11_H_6_O_3_	P	[M + H]^+^
Peiminine	C_27_H_43_NO_3_	P	[M + H]^+^
Peimine	C_27_H_45_NO_3_	P	[M + H]^+^
Norharman	C_11_H_8_N_2_	P	[M + H]^+^
Myrcene	C_10_H_16_	P	[M + Na]^+^
Linolenic acid	C_18_H_30_O_2_	P	[M + H]^+^
Isopimpinellin	C_13_H_10_O_5_	P	[M + H]^+^
Chlorogenic acid	C_16_H_18_O_9_	P	[M + H]^+^
Catalpol	C_15_H_22_O_10_	P	[M + Na]^+^
Arctigenin	C_21_H_24_O_6_	P	[M + NH_4_]^+^
Adenosine	C_10_H_13_N_5_O_4_	P	[M + H]^+^
_5_-methoxypsoralen	C_12_H_8_O_4_	P	[M + H]^+^
Valine	C_5_H_11_NO_2_	P	[M + H]^+^
Proline	C_5_H_9_NO_2_	P	[M + H]^+^
Luteolin	C_15_H_10_O_6_	P	[M + H]^+^
Kaempferol	C_15_H_10_O_6_	P	[M + H]^+^
Hesperidin	C_28_H_34_O_15_	P	[M + H]^+^
Choline	C_5_H_14_NO	P	[M]^+^
Amentoflavone	C_30_H_18_O_10_	P	[M + H]^+^
Diosmetin	C_16_H_12_O_6_	P	[M + H]^+^
Inosine	C_10_H_12_N_4_O_5_	P	[M + Na]^+^
DL-praeruptorin A	C_21_H_22_O_7_	P	[M + Na]^+^

**TABLE 4:** The blood-absorbed components of QRHTLQ.

Metabolite name	Formula	Ion	Adduct type
Wogonin	C_16_H_12_O_5_	P/N	[M + H]^+^, [M-H]^-^
Vanillic acid	C_8_H_8_O_4_	P	[M + Na]^+^
Umbelliferone	C_9_H_6_O_3_	P	[M + H]^+^
Trigonelline	C_7_H_7_NO_2_	P	[M + H]+
Skullcapflavone II	C_19_H_18_O_8_	P/N	[M + H]^+^, [M-H]^-^
Salidroside	C_14_H_20_O_7_	P	[M + H]^+^
Psoralen	C_11_H_6_O_3_	P	[M + H]^+^
Phenylalanine	C_9_H_11_NO_2_	P/N	[M + H]^+^, [M-H]^-^
Peimisine	C_27_H_41_NO_3_	P	[M + H]^+^
Peiminine	C_27_H_43_NO_3_	P	[M + H]^+^
Peimine	C_27_H_45_NO_3_	P	[M + H]^+^
Norharman	C_11_H_8_N_2_	P	[M + H]^+^
Myrcene	C_10_H_16_	P	[M + Na]^+^
Linoleic acid	C_18_H_32_O_2_	P	[M + H]^+^
Inosine	C_10_H_12_N_4_O_5_	P/N	[M + Na]^+^, [M-H]^-^
DL-praeruptorin A	C_21_H_22_O_7_	P	[M + Na]^+^
Diosmetin	C_16_H_12_O_6_	P	[M + H]^+^
Baicalein	C_15_H_10_O_5_	P/N	[M + H]^+^, [M-H]^-^
Adenosine	C_10_H_13_N_5_O_4_	P	[M + H]^+^
Choline	C_5_H_14_NO^+^	P	[M]^+^
Proline	C_5_H_9_NO_2_	P	[M + H]^+^
Valine	C_5_H_11_NO_2_	P/N	[M + H]^+^, [M-H]^-^
Baicalin	C_21_H_18_O_11_	P/N	[M + H]^+^, [M-H]^-^
Ursolic acid	C_30_H_48_O_3_	N	[M-H]^-^
Succinic acid	C_4_H_6_O_4_	N	[M-H]^-^
Sinapyl alcohol	C_11_H_14_O_4_	N	[M-H]^-^
Palmitic acid	C_16_H_32_O_2_	N	[M-H]^-^
Oroxindin	C_22_H_20_O_11_	N	[M-H]^-^
Oleic acid	C_18_H_34_O_2_	N	[M-H]^-^
Catechol	C_6_H_6_O_2_	N	[M-H]^-^
Adenine	C_5_H_5_N_5_	N	[M-H]^-^

### 3.4 A network pharmacology analysis identifies MAPK14 and IL-6 as core targets of QRHTLQ in the treatment of FPM-Induced pulmonary injury

**FIGURE 4:**
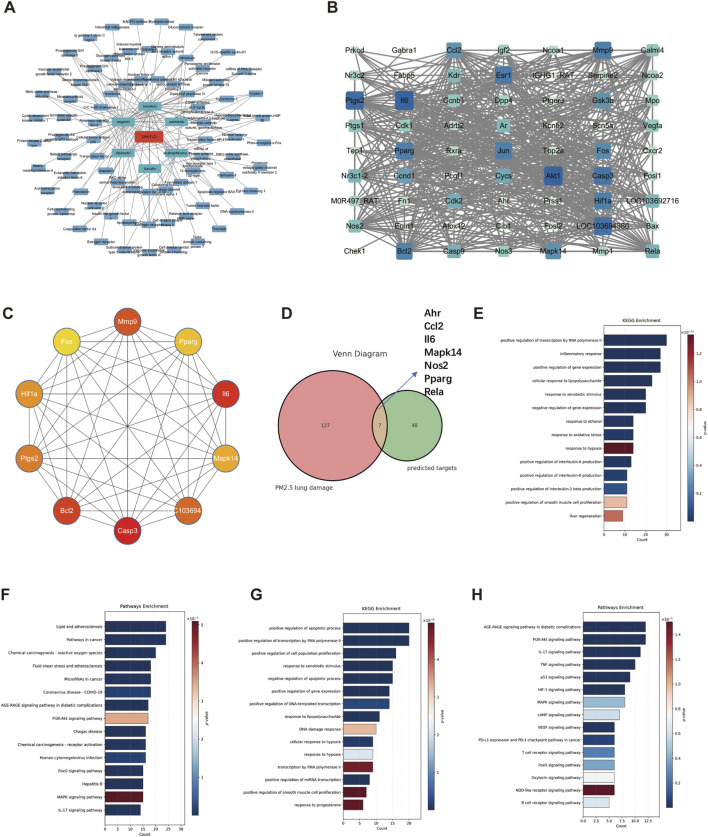
Network pharmacology suggested that MAPK14 is a potential key target for QRHTLQ in intervening FPM-induced lung injury. **(A)** Predicted targets of the main blood-absorbed components of QRHTLQ obtained using the TCMSP database. **(B)** Weighted protein‒protein interaction (PPI) network constructed using Cytoscape. **(C)** Screen of the top 10 core proteins using Cytoscape. **(D)** Venn diagram of FPM disease-related proteins and predicted targets. **(E,F)** Results of the enrichment analysis of proteins related to FPM-induced disease. **(G,H)** Results of the enrichment analysis of the predicted targets of QRHTLQ.

We predicted the potential mechanisms by which the primary active components of QRHTLQ intervene in FPM-induced lung injury using network pharmacology. Targets of the major active blood-absorbed constituents of QRHTLQ were screened using the TCMSP database (Figure 4A), and protein–protein interactions (PPIs) were analyzed using STRING, followed by weighted visualization with Cytoscape 3.10 (Figure 4B). Key nodes with high degree values were identified, including *Il-6*, *Mapk14*, *Akt1*, *Mmp9*, *Jun*, and *Caspase3*, suggesting that these molecules may serve as core targets of QRHTLQ (Figure 4C). Gene targets associated with FPM-induced lung injury were retrieved from GeneCards and intersected with QRHTLQ-predicted targets, resulting in seven overlapping targets: *Ahr, Il-6, Ccl2, Mapk14, Nos2, Pparg,* and *Rela* (Figure 4D). A subsequent enrichment analysis of both FPM-related genes (Figures 4E,F) and QRHTLQ-associated targets (Figures 4G,H) revealed biological processes and pathways involved in inflammatory responses and oxidative stress. Notably, both analyses consistently highlighted IL-6 and the MAPK signaling pathway, indicating that IL-6 and MAPK14 are likely pivotal targets that mediate the protective effects of QRHTLQ on FPM-induced lung injury.

### 3.5 Proteomic analysis suggests that QRHTLQ May inhibit FPM-Induced NETs formation

**FIGURE 5:**
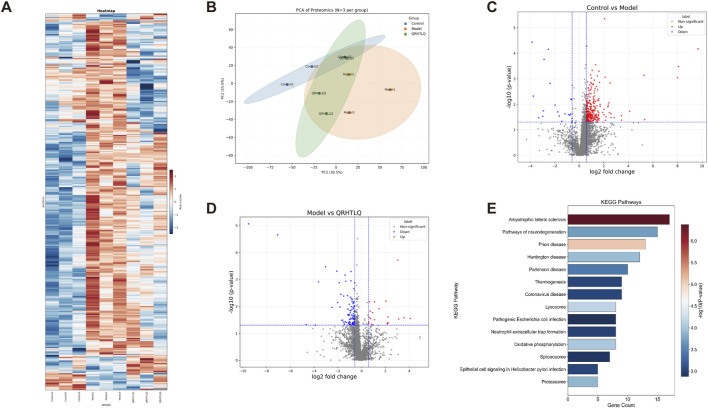
Proteomic analysis suggested that QRHTLQ may alleviate FPM-induced lung injury by inhibiting NETs formation. **(A)** Heatmap of protein expression levels. **(B)** PCA of the samples. **(C)** Volcano plot of differentially expressed proteins between the control group and FPM model group. **(D)** Volcano plot of differentially expressed proteins between the QRHTLQ-M group and model group. **(E)** Results of the enrichment analysis of differentially expressed proteins between the QRHTLQ-M group and model group.

To further explore the mechanisms underlying the protective effects of QRHTLQ, we performed proteomic analysis on three groups: the Control, Model, and QRHTLQ-M groups. The principal component analysis visualized using Python revealed clear heterogeneity among the experimental groups (Figure 5B). Differentially expressed proteins were subsequently identified and visualized (Figures 5C,D).

The enrichment analysis of differentially expressed proteins between the QRHTLQ medium-dose group and the model group indicated that QRHTLQ may modulate biological processes related to the formation of NETs (Figure 5E). Previous studies have shown that FPM exposure can induce NETs formation in lung tissue. Based on this evidence, we focused on NETs formation as a key biological process for further validation and investigation.

### 3.6 QRHTLQ inhibits FPM-induced NET formation in lung tissue

**FIGURE 6:**
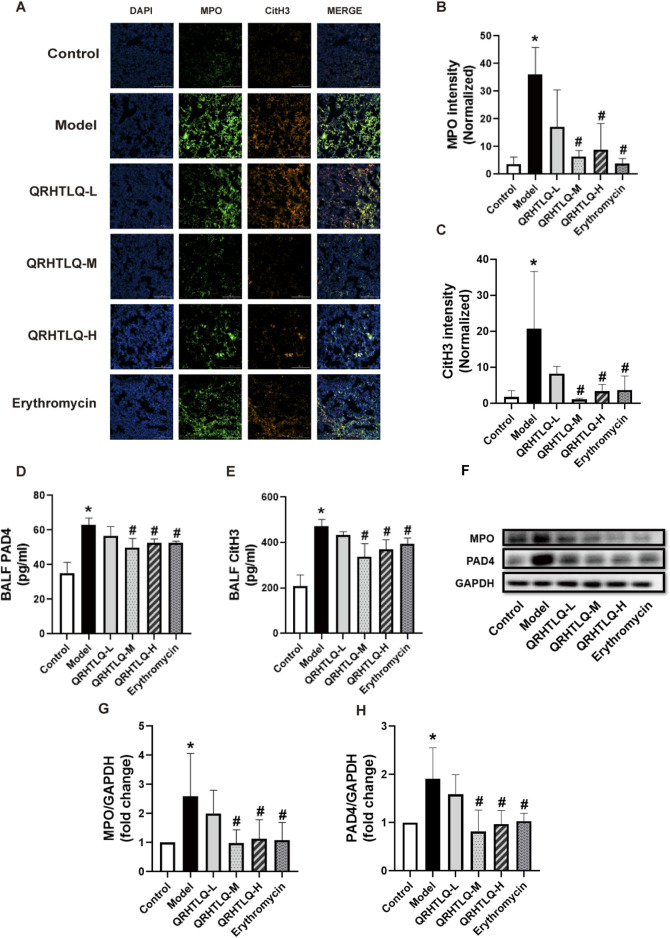
QRHTLQ attenuates FPM-induced NETs formation in the lungs. **(A)** Dual immunofluorescence staining for MPO and CitH3. **(B,C)** Quantification of MPO and CitH3 fluorescence intensity, respectively (n = 3) **(D,E)** Contents of PAD4 and CitH3 in the BALF from each group (n = 5). **(F)** Representative images of MPO and PAD4 levels detected by Western blotting. **(G,H)** Statistical analysis of the gray values of MPO and PAD4 (n = 5). Compared with the model group: #*p* < 0.05. Compared with the control group: *p < 0.05.

CitH3 and MPO are the core components of NETs. Dual immunofluorescence staining for CitH3 and MPO was performed to evaluate NETs formation following FPM exposure and the QRHTLQ intervention. Immunofluorescence analysis revealed that FPM exposure induced strong green fluorescence of MPO and robust orange-red fluorescence of CitH3 with distinct spatial colocalization in lung tissues, indicating active *in vivo* NETs formation. However, QRHTLQ treatment significantly diminished the fluorescence intensities (Figures 6A–C, *p* < 0.05).

### 3.7 QRHTLQ reduces the FPM-induced upregulation of PAD4 and MPO

PAD4 is a key enzyme involved in NETs formation. Upon stimulation, it becomes activated and translocates into the nucleus, where it catalyzes the citrullination of histones, leading to chromatin decondensation and subsequent NETs release. The levels of PAD4 in BALF and lung tissue were measured using ELISA and WB, respectively, to assess the inhibitory effect of QRHTLQ on this pathway.

The ELISA results showed that the intratracheal instillation of FPM significantly increased PAD4 and CitH3 levels in the BALF, whereas QRHTLQ treatment effectively reduced this increase (Figures 6D,E). Western blot results further confirmed that FPM exposure markedly upregulated PAD4 and MPO expression in lung tissue, whereas QRHTLQ administration significantly attenuated this increase (Figures 6F–H). These results indicate that QRHTLQ significantly attenuates the FPM-induced increase in pulmonary NETs, and the underlying mechanism may be closely related to the downregulation of PAD4 expression.

### 3.8 QRHTLQ decreases the pulmonary p-P38/P38 ratio

**FIGURE 7:**
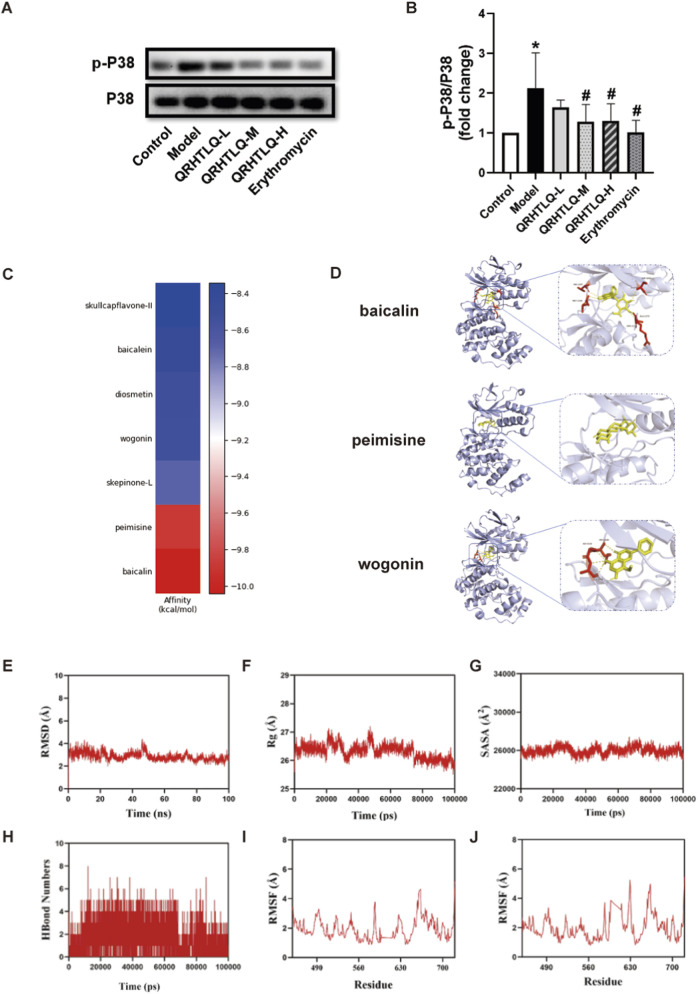
Baicalin may act as a potential inhibitor of MAPK14 within QRHTLQ. **(A)** Representative images of p-P38 and P38MAPK levels in lung tissues detected by Western blotting. **(B)** Statistical graph of the p-P38/P38MAPK ratio (n = 5). **(C)** Heatmap of molecular docking between the main active blood-absorbed components of QRHTLQ and MAPK14. **(D)** Visualization of the molecular docking results. **(E)** RMSD values of protein–ligand complexes over time. **(F)** Rg values of protein–ligand complexes over time. **(G)** SASA values of protein–ligand complexes over time. **(H)** H bond values of protein–ligand complexes over time. **(I,J)** RMSF values of amino acid backbone atoms in protein–ligand complexes over time. Compared with the model group: #*p* < 0.05. Compared with the control group: *p < 0.05.

Network pharmacology analysis indicated that MAPK14 may serve as a key target through which QRHTLQ exerts its protective effects on FPM-induced lung injury. Given that the MAPK/P38 pathway has been reported to promote NETosis, we further investigated the regulatory effect of QRHTLQ on MAPK14 activity. WB was performed to detect the relative levels of p-P38 and P38 in lung tissue. The intratracheal instillation of FPM significantly increased the p-P38/P38 ratio, whereas QRHTLQ treatment reduced this ratio in a dose-dependent manner (Figures 7A,B).

### 3.9 Molecular docking and molecular dynamics simulation suggest baicalin as a potential inhibitor of MAPK14

To explore the molecular basis underlying the reduction in the p-P38/P38 ratio in lung tissue induced by QRHTLQ, we performed molecular docking of the major blood-absorbed active components of QRHTLQ with MAPK14. Skepinone-L, a known MAPK14 inhibitor, was included as a reference compound. The docking results revealed that baicalin exhibited the lowest binding energy (−10.04 kcal/mol), ranking first among all the tested compounds (Figures 7C,D), including skepinone-L, suggesting that baicalin may serve as a potential inhibitor of MAPK14.

To further examine this interaction, we performed molecular dynamics simulations for the baicalin–MAPK14 complex. The system reached equilibrium after 20 ns, and the RMSD fluctuated around 2.5 Å thereafter (Figure 7E). The radius of gyration (Rg) and solvent-accessible surface area (SASA) exhibited slight fluctuations during the simulation, indicating conformational adjustments within the complex (Figures 7F,G). The complex maintained approximately three to five hydrogen bonds throughout most of the simulation (Figure 7H), and the RMSF values were relatively low (predominantly below 3 Å), reflecting low flexibility and high stability (Figures 7I,J).

Taken together, these results indicate that the baicalin–MAPK14 complex displays stable binding interactions with favorable hydrogen bonding, supporting baicalin as a promising small-molecule inhibitor targeting P38.

### 3.10 QRHTLQ-containing serum attenuates FPM-induced NETs formation in dHL-60 cells

**FIGURE 8:**
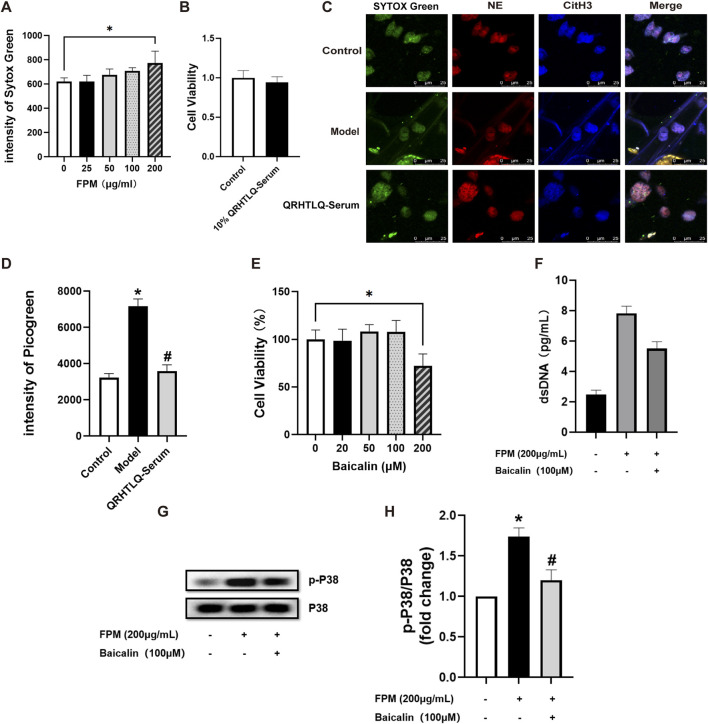
QRHTLQ-containing serum and baicalin inhibit FPM-induced extracellular dsDNA release, NETs formation, and p38 MAPK pathway activation in dHL-60 cells. **(A)** SYTOX Green fluorescence intensity detecting extracellular dsDNA release in dHL-60 cells stimulated with different concentrations of FPM (0, 25, 50, 100, 200 μg/mL) for 4 h. **(B)** Effect of 10% QRHTLQ-containing serum on dHL-60 cell viability. **(C)** Immunofluorescence staining showing NETs formation in FPM-stimulated dHL-60 cells (green, SYTOX Green; red, NE; blue, CitH3). **(D)** Relative dsDNA fluorescence intensity in cell supernatants detected by PicoGreen after 4 h of FPM stimulation. **(E)** Effects of different concentrations of baicalin (0, 20, 50, 100, 200 μM) on dHL-60 cell viability. **(F)** Inhibitory effect of baicalin (100 μM) on FPM (200 μg/mL)-induced dsDNA release (absolute quantification). **(G,H)** Western blot analysis of p-P38 and P38 protein levels and quantitative analysis of the p-P38/P38 ratio in each group (n = 3) Compared with the model group: #*p* < 0.05. Compared with the control group: *p < 0.05.

We used the dHL-60 cells (obtained by differentiating HL-60 cells in medium supplemented with 1.25% DMSO for 5 days) to investigate the effect of FPM on NETs formation. The DNA component of NETs was quantitatively measured via SYTOX Green assay to reflect NETs production in dHL-60 cells under different stimulation conditions. The results showed that 200 μg/mL FPM could markedly induce NETs formation after 4 h stimulation (Figure 8A).

The CCK-8 assay showed that 10% QRHTLQ-containing serum had no significant effect on the viability of dHL-60 cells (Figure 8B). Therefore, we selected 10% QRHTLQ-containing serum as the working concentration to investigate its intervention on NETs formation. Subsequent results demonstrated that stimulation with 200 μg/mL FPM significantly increased the expression of NE and CitH3 in and around the cells, and the two proteins exhibited obvious co-localization, indicating that 200 μg/mL FPM could significantly induce NETs formation in dHL-60 cells (Figure 8C). Immunofluorescence observations suggested that QRHTLQ-containing serum exhibited a trend toward inhibiting NETs formation. Concurrently, we used the PicoGreen assay kit to measure the fluorescence intensity of dsDNA in the cell culture supernatants of each group (Figure 8D). The results demonstrated that QRHTLQ-containing serum could inhibit the FPM-induced release of NETs.

### 3.11 Baicalin interferes with NETs formation and regulates the p-P38/P38 ratio in dHL-60 cells

The CCK-8 assay showed that baicalin at concentrations up to 100 μM had no significant effect on the viability of dHL-60 cells (Figure 8E).

We used a dsDNA kit to quantitatively measure the dsDNA content in the supernatant of each group. The results showed that 200 μg/mL FPM significantly increased the dsDNA level in the culture supernatant of dHL-60 cells. Pretreatment of dHL-60 cells with 100 μM baicalin for 2 h decreased the dsDNA production induced by FPM stimulation. The above results suggest that 100 μM baicalin can significantly interfere with NETs formation (Figure 8F).

Meanwhile, we performed Western blot to examine the activation of MAPK14 in the cell pellets of each group. Stimulation of dHL-60 cells with 200 μg/mL FPM for 4 h significantly increased the p-P38/P38 ratio, indicating the activation of MAPK14. Pretreatment of dHL-60 cells with 100 μM baicalin for 2 h significantly decreased the p-P38/P38 ratio, suggesting that baicalin can inhibit the activation of MAPK (Figures 8G,H).

## 4 Discussion

Our previous studies have shown that QRHTLQ can relieve the symptoms of patients with AECOPD and attenuate AECOPD induced by an intratracheal instillation of LPS combined with cigarette smoke exposure via the EGFR–PI3K–AKT signaling pathway (Feng et al., 2019). However, whether QRHTLQ can mitigate FPM-induced lung injury and the underlying mechanisms remain unclear. In the present study, we found that QRHTLQ alleviated FPM-induced lung injury by reducing inflammatory cell infiltration in the airways, decreasing the mRNA expression of proinflammatory cytokines, and suppressing the formation of NETs. In addition, baicalin, one of the major blood-absorbed constituents of QRHTLQ, formed a stable complex with MAPK14 and may represent one of the key active substances mediating the effects of QRHTLQ.

QRHTLQ decreased the mRNA levels of inflammatory cytokines in lung tissue and reduced ROS levels in BALF, thereby exerting a protective effect on FPM-induced lung injury. We investigated the protective effects of QRHTLQ in a lung injury model induced by intratracheal instillation of FPM. H&E staining and qPCR analysis showed that QRHTLQ alleviated inflammatory cell infiltration in the lungs and downregulated the mRNA expression of IL-1β, IL-6 and TNF-α. Previous studies have indicated that FPM in lungs triggers intense oxidative stress and excessive production of ROS (Wang et al., 2017; Hamanaka and Mutlu, 2025). The accumulation of ROS not only directly causes oxidative damage to cell membranes but also acts as a key signaling molecule that activates downstream inflammation-related pathways, leading to further ROS production and tissue injury. In our study, we observed that FPM exposure increased ROS levels in rat BALF, whereas QRHTLQ reversed this change. These findings suggest that QRHTLQ can interrupt the vicious cycle of the excessive production of ROS and inflammatory mediators, thereby attenuating FPM-induced lung injury.

To further elucidate the underlying mechanisms, we analyzed the differentially expressed proteins between the Model and QRHTLQ groups. The enrichment analysis suggested that the formation of NETs may be a pivotal pathological target for QRHTLQ. While NETs are an indispensable part of the host defense system, their excessive formation in response to environmental pollutants like FPM can trigger the release of cytotoxic components, thereby exacerbating lung tissue injury (Scozzi et al., 2022; Xuan et al., 2023). Our findings demonstrate that QRHTLQ significantly suppressed the FPM-induced elevation of pulmonary NETs and effectively downregulated key contributors to the NETosis process, including the essential enzymes PAD4 and MPO (Metzler et al., 2011; Mauracher et al., 2018). The significance of these results lies in the fact that the protective effect of QRHTLQ against FPM-induced lung injury is not merely a generalized inhibition of inflammatory cytokines. Instead, it exerts its therapeutic potential through multi-level modulation of neutrophil activation and homeostasis. This provides experimental evidence for the use of Traditional Chinese Medicine formulas in counteracting environmental stress-induced damage by maintaining neutrophil functional stability.

The potential molecular link between QRHTLQ and the observed reduction in NETs was further illuminated by our network pharmacology analysis, which identified MAPK14 as a core target. Among various MAPK isoforms, MAPK14 is highly responsive to oxidative stress, acting as a pivotal transducer of redox signals. The biochemical significance of this target lies in its role within the MAPK14–PAD4 axis; previous evidence has established that ROS-induced activation of p-P38 facilitates the expression of PAD4, the requisite enzyme for chromatin decondensation in NETosis. By demonstrating a significant decrease in the p-P38/P38 ratio *in vivo*, our results align with the hypothesis that QRHTLQ mitigates aberrant NETs generation by deactivating this oxidative-sensitive signaling axis. This is consistent with earlier reports where P38 MAPK inhibition successfully suppressed NETs formation in other inflammatory models, such as those induced by LPC, thereby reinforcing the role of MAPK14 as a viable therapeutic node for QRHTLQ (Liu et al., 2024).

Our results indicate that baicalin is a potential inhibitor of MAPK14. *In vitro* experiments of this study found that baicalin reduced the release of extracellular dsDNA in dHL-60 cells and inhibited the activation level of MAPK14, the target protein screened in our preliminary work. Previous studies have confirmed that MAPK14 is significantly activated in FPM-induced neutrophilic inflammation, which is consistent with our observations. Meanwhile, baicalin markedly downregulated MAPK14 activation, further suggesting that baicalin can intervene in FPM-induced lung injury by regulating the MAPK14–PAD4–NETs signaling axis. Our research broadens the pharmacological application scope of baicalin, further elucidates the potential molecular mechanism of its therapeutic effects, and provides new perspectives for further in-depth and comprehensive follow-up studies.

Currently, a major bottleneck in environmental lung injury research is that most therapeutic strategies focus on the management of complications after disease onset or generalized anti-inflammatory therapy at the first sign of symptoms. Consequently, there is a distinct lack of early-stage preventive intervention guided by the TCM philosophy of ‘preventive treatment of disease’, as well as a lack of targeted functional regulation of neutrophils. This study initiates early intervention from the onset of environmental exposure and identifies the inhibition of excessive NETs formation as one important biological process involved in the protective effects of QRHTLQ. These findings provide experimental evidence in the early prevention and treatment of environment-induced lung injury.

From the perspective of TCM theory, FPM-induced lung injury may be interpreted as a process in which external haze toxin invades the lung, transforms into heat, promotes phlegm accumulation, and impairs the diffusion and descent of lung Qi. Therefore, the core pathogenesis can be summarized as toxic heat, phlegm obstruction, and lung Qi stagnation. QRHTLQ is designed to clear heat, resolve phlegm, regulate Qi, and relieve wheezing. From a modern biological perspective, its effects on oxidative stress, inflammatory mediators, MAPK14 activation, and NETs formation may partially explain the biological basis of these traditional therapeutic principles.

In summary, our study revealed that (1) QRHTLQ reduces the expression of inflammatory cytokines in lung tissue and suppresses excessive oxidative stress; (2) QRHTLQ decreases FPM-induced pulmonary NETs formation; and (3) QRHTLQ inhibits the FPM-induced overactivation of MAPK14, and one of the blood-absorbed components of QRHTLQ, baicalin, is a potential inhibitor of MAPK14.

Although this study reported the efficacy of QRHTLQ in protecting against FPM-induced lung injury, we also recognize several limitations: (1) we used a relatively short-term, high-dose FPM exposure model, which differs from the real-world scenario of long-term, low-dose exposure in human populations; (2) we focused primarily on NETs formation, as suggested by proteomics and network pharmacology, while other important processes and signaling pathways remain to be explored more broadly; and (3) in this study, we confirmed that QRHTLQ and baicalin could inhibit FPM-induced MAPK14 activation mainly through computer simulation analysis as well as *in vivo* and *in vitro* experiments; nevertheless, its specificity remains to be further verified by gene-editing technology.

## 5 Conclusion

Taken together, the results of the present study suggest that QRHTLQ attenuates FPM-induced lung injury by alleviating excessive oxidative stress, reducing the mRNA expression of inflammatory cytokines in lung tissue, and suppressing the formation of neutrophil extracellular traps. Moreover, the MAPK14/NETs axis may represent one of the key biological processes involved in the protective effects of QRHTLQ, with baicalin serving as a potential active constituent associated with MAPK14 regulation. These findings support the multitarget and multiprocess therapeutic effects of QRHTLQ as a traditional Chinese herbal formula, provide a basis for its broader clinical application, and offer support for the role of traditional Chinese medicine in preventing and treating environmental pollutant-induced tissue injury.

## Data Availability

The data presented in the study are deposited in the iProX repository, accession number PXD079805.

## Glossary

AECOPD: acute exacerbation of chronic obstructive pulmonary disease
BALF: bronchoalveolar lavage fluid
BCA: bicinchoninic acid
cDNA: complementary DNA
CHARMM36: Chemistry at HARvard Macromolecular Mechanics 36 force field
CitH3: citrullinated histone H3
COPD: chronic obstructive pulmonary disease
DAVID: Database for Annotation, Visualization and Integrated Discovery
DL: drug likeness
EGFR: epidermal growth factor receptor
ELISA: enzyme-linked immunosorbent assay
FDR: false discovery rate
FPM: particulate matter with aerodynamic diameter ≤2.5 μm
GAPDH: glyceraldehyde-3-phosphate dehydrogenase
GROMACS: GROningen MAchine for Chemical Simulations
H&E: hematoxylin and eosin
IL-1β: interleukin-1 beta
IL-6: interleukin-6
LC–MS: liquid chromatography–mass spectrometry
LPS: lipopolysaccharide
MAPK: mitogen-activated protein kinase
MAPK14: mitogen-activated protein kinase 14 (P38α)
MD: molecular dynamics
MPO: myeloperoxidase
MS/MS: tandem mass spectrometry
NETs: neutrophil extracellular traps
OB: oral bioavailability
PAD4: peptidylarginine deiminase 4
PBS: phosphate-buffered saline
PCA: principal component analysis
PPI: protein–protein interaction
PVDF: polyvinylidene difluoride
qPCR: quantitative polymerase chain reaction
QRHTLQ: Qingre–Huatan–Liqi formula
RIPA: radioimmunoprecipitation assay
RNA: ribonucleic acid
ROS: reactive oxygen species
SDS–PAGE: sodium dodecyl sulfate–polyacrylamide gel electrophoresis
SPF: specific pathogen-free
SRM: Standard Reference Material
STRING: Search Tool for the Retrieval of Interacting Genes/Proteins
TCMSP: Traditional Chinese Medicine Systems Pharmacology (database)
TNF-α: tumor necrosis factor alpha
TRIzol: TRIzol reagent
UniProt: Universal Protein Resource
